# Metformin promotes cholesterol efflux in macrophages by up-regulating FGF21 expression: a novel anti-atherosclerotic mechanism

**DOI:** 10.1186/s12944-016-0281-9

**Published:** 2016-06-21

**Authors:** Fei Luo, Yuan Guo, Guiyun Ruan, Xiangping Li

**Affiliations:** Department of Internal Cardiovascular Medicine, The Second Xiangya Hospital of Central South University, 139 Middle Renmin Road, Changsha, 410011 People’s Republic of China

**Keywords:** Metformin, FGF21, Macrophage, ABCA1, ABCG1, Cholesterol efflux

Metformin is widely used as a glucose-lowering agent in patients with type 2 diabetes (T2D). Recently, several studies have confirmed that metformin possessed multiple cardiovascular protective effects such as decreasing cardiovascular mortality, attenuating adverse cardiac and vascular remodeling and ameliorating atherosclerosis [1–3]. Hong et al. [4] in a multicenter, randomized, double-blind, placebo-controlled clinical trial found treatment with metformin for 3 years substantially reduced major cardiovascular events in a median follow-up of 5.0 years compared with glipizide in type 2 diabetes patients who had a history of coronary artery disease. Forouzandeh et al. [5] demonstrated metformin treatment significantly attenuated high-fat diet-induced atherosclerosis in apolipoprotein E-knockout (*ApoE*^−/−^) mice. These studies suggest metformin could ameliorate atherosclerosis, but the anti-atherosclerotic mechanism of metformin remains to be fully clarified.

Previous studies reported that metformin could decrease plasma cholesterol levels in diabetic and non-diabetic patients beyond its glucose-lowering effect [6–9], and the lipid-lowering effect of metformin was also confirmed in a meta-analysis of randomized controlled trials (RCTs) [10]. Besides, it was found that metformin treatment could lower inflammation markers levels in patients at high risk of cardiovascular disease and restore impaired HDL-mediated cholesterol efflux from macrophages due to glycation [11, 12]. These effects may contribute to the anti-atherosclerotic properties of metformin.

The deposition of excessive cholesterol in macrophages plays a key role in atherosclerotic plaque formation, thus removal of excess cholesterol from macrophages may attenuate and even regress the atherosclerosis [13]. It has been identified that removal of cholesterol from macrophages was mediated by several transmembrane transporters, including adenosine triphosphate binding cassette (ABC) transporters A1 and G1 [14]. Yvan-Charvet et al. [15] evidenced that combined deficiency of Abca1 and Abcg1 in macrophages severely damaged cholesterol efflux and accelerated atherosclerosis in low-density lipoprotein receptor-knockout (*Ldlr*^-/-^) mice. Bochem et al. [16] demonstrated that carriers of *ABCA1* loss-of-function mutations presented with lower cholesterol efflux capacity and higher atherosclerotic burden compared with age and sex-matched controls. Conversely, increased expression of ABCA1 and ABCG1 was involved in redistribution of cholesterol from inner to outer leaflet of the plasma membrane, facilitating cholesterol efflux from cholesterol-loaded macrophages [17]. Overexpression of *ABCA1* in macrophages increased cholesterol efflux by about 60 % (*P* = 0.0006) and reduced atherosclerotic plaques by about 68 % (*P* = 0.0008) in *Ldlr*^-/-^ mice fed a Western-type diet for 12 weeks [18]. Thus, ABCA1- and ABCG1-driven cholesterol efflux in macrophages was a key regulator in anti-atherosclerosis.

Fibroblast growth factor (FGF) 21, a member of FGF subfamily and mainly secreted by liver and adipose tissue, is a novel metabolic regulator with multiple cardioprotective efficacy [19]. It was found that FGF21 deficiency caused a marked exacerbation of atherosclerotic plaque formation in *ApoE*^−/−^ mice [20]. Moreover, previous studies also demonstrated that FGF21 could activate ABCA1 and ABCG1 expression of macrophages and in turn promoted cholesterol efflux in macrophages [21, 22], suggesting up-regulation of FGF21 exerted function of reducing atherosclerotic plaques. Intriguingly, Nygaard et al. [23] found metformin significantly increased FGF21 expression in both rat and human hepatocytes. Another study showed metformin could increase serum FGF21 levels both in mice and human [24]. Thus, we speculate metformin stimulates ABCA1 and ABCG1 expression via up-regulation of FGF21.

Taken together, we hypothesize metformin increases FGF21 expression, and subsequently promotes expression of ABCA1 and ABCG1 in macrophages, which promotes cholesterol efflux from macrophages and attenuated atherosclerotic plaques. Further exploring cardioprotective mechanism of metformin will be useful for insight into novel therapeutic targets.

## Abbreviations

ABC, adenosine triphosphate binding cassette; ABCA1, adenosine triphosphate binding cassette transporters A1; ABCG1, adenosine triphosphate binding cassette transporters G1; ApoE, apolipoprotein E; FGF, fibroblast growth factor; FGF21, fibroblast growth factor 21; Ldlr, low-density lipoprotein receptor; RCT, randomized controlled trial; T2D, type 2 diabetes.
